# Beyond Breast and Ovarian Cancers: PARP Inhibitors for BRCA Mutation-Associated and BRCA-Like Solid Tumors

**DOI:** 10.3389/fonc.2014.00042

**Published:** 2014-02-28

**Authors:** Ciara C. O’Sullivan, Dominic H. Moon, Elise C. Kohn, Jung-Min Lee

**Affiliations:** ^1^Medical Oncology Branch, Center for Cancer Research, National Cancer Institute, Bethesda, MD, USA; ^2^Medical Research Scholars Program, National Institutes of Health, Bethesda, MD, USA

**Keywords:** poly(ADP-ribose) polymerase inhibitors, solid tumors, BRCA mutation, BRCA-like, DNA damage repair pathway

## Abstract

Poly(ADP-ribose) polymerase inhibitors (PARPi) have shown clinical activity in patients with germline BRCA1/2 mutation (gBRCAm)-associated breast and ovarian cancers. Accumulating evidence suggests that PARPi may have a wider application in the treatment of cancers defective in DNA damage repair pathways, such as prostate, lung, endometrial, and pancreatic cancers. Several PARPi are currently in phase I/II clinical investigation, as single-agents and/or combination therapy in these solid tumors. Understanding more about the molecular abnormalities involved in BRCA-like phenotype in solid tumors beyond breast and ovarian cancers, exploring novel therapeutic trial strategies and drug combinations, and defining potential predictive biomarkers are critical to expanding the scope of PARPi therapy. This will improve clinical outcome in advanced solid tumors. Here, we briefly review the preclinical data and clinical development of PARPi, and discuss its future development in solid tumors beyond gBRCAm-associated breast and ovarian cancers.

## Introduction

Increasing understanding of the cellular aberrations inherent to cancer cells has allowed the development of therapies targeting biological pathways. This approach has been an important step toward individualization of therapy for germline BRCA1/2 mutation (gBRCAm)-associated breast and ovarian cancers (1, 2). The clinical development of poly(ADP-ribose) polymerase inhibitors (PARPi), with their selective mechanisms of action involving the DNA damage repair pathways, is an example of this strategy. Early clinical trials have shown significant single-agent activity of PARPi in gBRCAm-associated breast and ovarian cancers (3–5). Response rates (RR) of 31–40% have been reported in gBRCAm ovarian cancer patients with measureable recurrent disease, and the RR and duration of response to PARPi monotherapy has been associated with platinum sensitivity (6, 7). Emerging evidence suggests that PARPi is an effective therapeutic strategy in subsets of other malignancies that have gBRCAm, such as melanoma, prostate, and pancreatic cancers. BRCA-like tumors have molecular and clinical characteristics in common with tumors occurring in patients with gBRCAm, which may have implications for PARPi-based therapy (8). Additionally, there is a potential therapeutic role for PARP inhibition in a wider subgroup of solid tumors that may have defective homologous recombination (HR) (9). Therefore, the utility of PARPi in other solid tumors is potentially greater than was previously envisioned (8).

PARPi have shown to enhance cytotoxicity in combination with DNA methylating agents (10, 11), topoisomerase inhibitors (12, 13), platinums (14, 15), alkylating agents (14), and radiation (16, 17) in numerous preclinical studies. These preclinical findings are being explored in clinical trials to elucidate the role of PARPi as chemo- and radiosensitizers in various tumor types (18). A large number of clinical trials are exploring the efficacy of combination strategies in malignancies such as non-small cell lung cancer (NSCLC), squamous cell cancer of the head and neck (HNSCC), esophageal, and colorectal cancers (CRCs) (Tables 1 and 2); the results of several phase I and II trials have already been reported (Table 3). These data suggest further clinical exploration of PARPi as monotherapy or combinations is warranted in patients not only with gBRCAm-associated breast or ovarian cancer, but also in solid tumors with HR dysfunction.

**Table 1:** **PARPi in clinical development (excluding breast and ovarian cancer) (19)**.

Name	Treatment	Cancer types	Phase
Olaparib (AstraZeneca)	Monotherapy	GBM, prostate, ES, NSCLC, CRC, and gastric cancer	I/II
	Combination with chemotherapy	Esophageal cancer and HNSCC	
	Combination with RT		
	Combination with targeted therapies		
Rucaparib (Clovis)	Combination with chemotherapy	AST	I
Veliparib (Abbott)	Monotherapy	gBRCAm prostate cancer, HNSCC, NSCLC, SCLC, pancreatic cancer, biliary cancers, HCC, rectal cancer, cervical cancer, CRPC, and CNS malignancies	I/II
	Combination with chemotherapy		
	Combination with RT		
	Combination with targeted therapies		
CEP-9722 (Cephalon)	Monotherapy	AST	I
	Combination with chemotherapy		
E7016 (EISAI)	Combination with chemotherapy	Melanoma and AST	I/II
BMN-673 (BioMarin)	Monotherapy	AST	I

*GBM, glioblastoma multiforme; ES, Ewing’s sarcoma; NSCLC, non-small cell lung cancer; SCLC, small cell lung cancer; CRC, colorectal cancer; AST, advanced solid tumors; HNSCC, head and neck squamous cell cancer; CRPC, castrate resistant prostate cancer; HCC, hepatocellular cancer; CNS, central nervous system; n/a, not applicable*.

**Table 2:** **Trials of PARPi in solid tumors (excluding breast and ovarian cancers)**.

Malignancy	PARPi	Combination agent(s)	Phase
**GI**			
Pancreatic	Olaparib	Chemotherapy	I/II
	Veliparib	Cisplatin	
		Gemcitabine	
		Gemcitabine/IMRT	
		Monotherapy (gBRCAm pancreatic cancer)	
		Modified FOLFOX 6	
Pancreatic, biliary, urothelial and NSCLC	Veliparib	Cisplatin and gemcitabine	I
Liver	Veliparib	Cisplatin and gemcitabine	I
Colorectal cancer	Veliparib	TMZ	I/II
	Olaparib	Irinotecan	
	Veliparib	Capecitabine and RT	
Colorectal cancer stratified by MSI	Olaparib	N/A	I/II
Esophageal cancer	Olaparib	RT	I
Gastric cancer	Veliparib	FOLFIRI	I/II
	Olaparib	Paclitaxel	
**LUNG**			
NSCLC (surgically unresectable)	Olaparib	Concurrent RT ± cisplatin	I/II
	Veliparib	RT	
		Carboplatin/paclitaxel	
		Cisplatin/gemcitabine	
EGFR mutation positive advanced NSCLC	Olaparib	Gefitinib ± olaparib	I/II
SCLC	Veliparib	Cisplatin/etoposide	I/II
		TMZ	
**GENITOURINARY**			
CRPC	Veliparib	Abiraterone and prednisone	I/II
		TMZ	
	Olaparib	N/A	II
**GYNECOLOGIC**			
Cervical cancer	Veliparib	Cisplatin and paclitaxel	I/II
		Topotecan	
		Carboplatin and paclitaxel	
Uterine carcinosarcoma	Veliparib	Carboplatin and paclitaxel	II
**CENTRAL NERVOUS SYSTEM**			
GBM	Olaparib	TMZ	I
	Veliparib	TMZ	I/II
Brain metastases	Veliparib	WBRT	I/II
DPG	Veliparib	RT	I/II
		TMZ	
Refractory CNS tumors	Veliparib	TMZ	I
**HEAD AND NECK**			
HNSCC	Veliparib	RT	I/II
		Docetaxel	
		5-FU	
**SARCOMA**			
Ewing’s sarcoma	Olaparib	N/A	II
**SKIN CANCER**			
Melanoma	Veliparib	TMZ	II
	E7016	TMZ	
**ADVANCED SOLID TUMORS**			
	Veliparib	Carboplatin and gemcitabine	I/II
		Gemcitabine	
		Carboplatin and paclitaxel	
		Mitomycin C	
		Capecitabine and oxaliplatin	
		Cyclophosphamide	
	Olaparib	Cisplatin/gemcitabine	
		PLD	
		Topotecan	
	Niraparib	Monotherapy	
	CEP-9722	Monotherapy	
	BMN-673	Monotherapy	

*IMRT, intensity modulated radiotherapy; NSCLC, non-small cell lung cancer; RT, radiotherapy; MSI, microsatellite instability; CRPC, castrate resistant prostate cancer; SCLC, small cell lung cancer; GBM, glioblastoma multiforme; DPG, diffuse pontine glioma; HNSCC, squamous cell carcinoma of the head and neck; 5-FU, 5-fluorouracil; PLD, pegylated liposomal doxorubicin*.

**Table 3:** **PARPi trials for which tumor response rates have been reported**.

PARPi	Patient cohort	Combination	Drug and schedule	Toxicity	Response
Rucaparib (20) (phase I)	AST	TMZ	D1: rucaparib 12 mg/m^2^ IV	No DLT	CR: 1/32 pts (melanoma)
	Melanoma (32 pts)		D1–5: TMZ 200 mg/m^2^ PO q 28 day cycle	Myelosuppression (13%)	PR: 2/32 pts (1 melanoma; 1 desmoid tumor)
				At MTD	SD: 7/32 pts-6 mo or greater
Olaparib (19) (phase I)	Melanoma (40 pts)	Dacarbazine	D1–7: olaparib (20–200 mg) PO BID	Grade 3 hypophosphatemia-1 pt	CR: 0/40 pts
			D1: (cycle 2 day 2): dacarbazine (600–800 mg/m^2^ IV) q 21 day cycle	Grade 3 neutropenia-1 pt Grade 4 neutropenia-2 pt	PR: 2/40 pts SD: 8/40 pts
			MTD: 100 mg olaparib PO BID and dacarbazine 600 mg/m^2^ IV		
Olaparib (21) (phase I)	AST (19 pts)	Topotecan	D1–3: topotecan 0.5–1.0 mg/m^2^ IV	DLTs 16%	CR: 0/19 pts
			Olaparib (50–200 mg PO BID) q 21 day cycle	Grade 3 thrombocytopenia-1 pt	PR: 1/19 pts
				Grade 4 neutropenia-2 pts	SD:4/19 pts
				Treatment related death-1 pt (pneumonia)	RECIST RR = 37%
Olaparib (22) (phase I)	AST (12 pts)	N/A	D1–28: olaparib (100–400 mg PO BID)	No DLTs	CR: 0/12
				Grade 3 toxicity in 16%	PR: 1/12 pts-13 mo
				Anemia-8%	SD: 4/12 > 8 weeks (unknown gBRCAm status)
				Elevated AST-8%	
Veliparib (23) (phase I)	AST (35 pts)	MCP	D1–21: cyclophosphamide 50 mg daily PO	DLTs-6%	CR: 0/35 pts
			Olaparib (20 mg daily × 7 days >80 mg daily q 21 days cycle) MTD: veliparib 60 mg daily and cyclophosphamide 50 mg once daily	Grade 3 ileus-1 pt	PR: 7/35 pts (gBRCAm)
				Grade 4 respiratory	SD: 6/35 (3 BRCA+)
				Failure and death-1 pt	
				Lymphopenia-34.3%	
INO-1001 (phase Ib)	Melanoma (12 pts)	TMZ	D1–5: TMZ 200 mg/m^2^ IV daily and INO-1001 (100–400 mg IV q 12 h) × 10 doses, q 28 day cycle MTD: INO-1001 = 400 mg	Anemia-17%	CR:0/12 pts
				Grade 4 hepatotoxicity-8%	PR:1/12 pts
				Grade 4 hematologic toxicity-58%	SD:4/12 pts
				Grade 3 myelosuppression	RR = 4.2%
					CBR = 41.6%
Rucaparib (24) (phase II)	Melanoma (40 pts)	TMZ	D1–5: TMZ 200 mg/m^2^ and rucaparib 12 mg/m^2^ IV, q 28 day cycle	Grade 4 thrombocytopenia-12%	10% PR (4/40 pts)
					SD-4/40 pts
Veliparib (25) (phase I/II)	Pancreatic cancer (18/28 pts evaluable at time of reporting)	Modified FOLFOX6	Phase 1 dose-escalation: veliparib 40–100 mg BID D1–7, q 14 day cycle	Grade 3 neutropenia-1 pt Grade 5 neutropenia-1 pt Grade 3 lymphopenia-1 pt Grade 3 anemia-1 pt	11 pts (First line) RR-18%
			Phase II-two parallel groups; first line and untreated		
					PFS-3.9 mo
					OS-7.4 mo
					Seven patients (pre-treated)
					RR-14%
					PFS-1.8 mo
					OS-5.4 mo
					CR-1/18 pts
					PR-1/18 pts
Veliparib (26) (phase II)	Colorectal cancer (47 pts)	TMZ	D1–5: TMZ 150 mg/m^2^ PO daily	Grade 3 A/E in 5 pts (myelosuppression)	ORR (CR + PR)-5%
			D1–7: veliparib 40 mg BID q 28-day cycle		2/47 pts-PR, 0/47 pts = CR
					CR + PR + SD = 23%
					Median TTP = 11 weeks; 23 weeks for pts with controlled disease

*DLT, dose limiting toxicity; MTD, maximum tolerated dose; CR, complete response; PR, partial response; SD, stable disease; IV, intravenously; PO, orally; D, days; mo, months; q, every; BID, twice a day; RECIST, response evaluation criteria in solid tumors; TMZ, temozolomide; AST, aspartate transaminase; pt(s), patient(s); MCP, metronomic cyclophosphamide; PFS, progression-free survival; OS, overall survival; ORR, overall response rate; TTP, time to progression*.

gBRCAm-associated and BRCA-like tumors are rare subsets of advanced solid tumors. Approximately 5–10% of breast (27) and 10–15% of ovarian cancers (28) occur in the setting of a hereditary cancer syndrome, the most common of which is a gBRCAm (29). This occurs less frequently in other solid tumors. Approximately 5% of cutaneous melanoma and gastric cancers are related to gBRCAm and 5–19% cases of familial pancreatic cancer are attributed to a gBRCAm (30, 31). Furthermore, gBRCAm are very rare events in patients with prostate cancer and NSCLC. gBRCAm are present in 0.44–1.2% of prostate cancer cases (32, 33). The overall incidence of gBRCAm in patients with NSCLC has not been reported from large trials; only 3 patients (2.7%) were noted to have a gBRCAm in a study of 110 Jewish men with epithelial growth factor receptor (EGFR) mutant-NSCLC (34). These subgroups of tumors with germline HR dysfunction constitute a rare population with recognized unmet therapeutic needs, and may be sensitive to treatment with PARPi. Additionally, there are significant unanswered questions of their use in solid tumors that have molecular and clinical characteristics in common with gBRCAm-associated tumors. Advances have been made in identifying new therapeutic targets and analyzing response to novel treatments in these patient subgroups and this has led to an explosion of PARPi-based clinical trials extending the patient cohort to include BRCA-like tumors.

## PARP Function and Inhibition in DNA Damage Repair Pathways

DNA damage can occur through various mechanisms from environmental factors such as ultraviolet rays, ionizing radiation, and genotoxic chemicals, to endogenous processes including generation of reactive oxygen species and replication (35). Highly complex and intertwined repair pathways have evolved to provide broad and redundant mechanisms to address damaged DNA: mismatch repair (MMR), base excision repair (BER), and nucleotide excision repair (NER) for a low fidelity single strand DNA break (SSB) repair mechanism, and HR and non-homologous end-joining (NHEJ) for double-strand DNA breaks (DSBs) (36). The different repair mechanisms are orchestrated by numerous enzymes to ensure the integrity of DNA essential for cell survival.

PARP are a family of enzymes that catalyze nicotinamide adenine dinucleotide (NAD^+^)-dependent ADP-ribosylation of DNA. PARP1 is the best characterized member of the PARP family, and PARP2 has a similar structure and function with varying affinity for substrates (37). PARP1 has been implicated in several DNA repair mechanisms including the repair of SSBs through the BER pathway. It recognizes and binds to DNA sites with SSB via its DNA binding domain, then subsequently synthesizes poly(ADP-ribose) (PAR) by transferring ADP-ribose molecules from NAD^+^ to itself and other acceptor proteins (38). This activates the formation of a DNA repair complex consisting of multiple repair proteins, including DNA ligase III and X-ray repair cross-complementing 1 (XRCC1) (39). The PARylated PARP1 dissociates from DNA as the negative charge of PAR decreases its affinity for DNA, and poly(ADP-ribose) glycohydrolase then degrades the PAR on PARP1 (40). PARP has been shown to have a direct involvement in DSB repair in addition to its role in preventing DSB formation by promoting BER. In PARP1-deficient cells, ATM-kinase function is compromised leading to a reduction in DNA DSB in response to radiation, indicating a role of PARP1 in ATM activation and HR (38, 41). PARP1 has been shown to reduce DSB formation by sensing stalled replication forks and recruiting MRE11 for end processing to initiate HR (42). Increased PARP1 expression and/or activity in tumor cells have been demonstrated in many tumor types (43, 44). Thus, HR dysfunction sensitizes cells to PARP inhibition leading to further chromosomal instability, cell cycle arrest, and apoptosis (45, 46).

**Figure 1:**
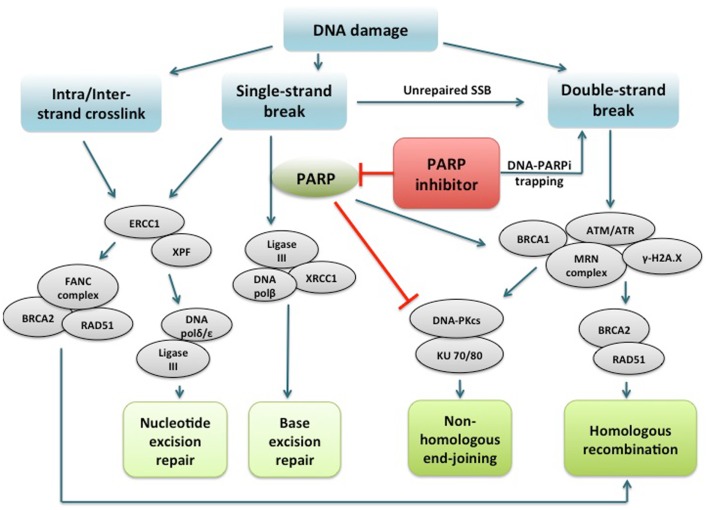
**PARP1 binds to DNA single strand break and catalyzes poly(ADP-ribosyl)ation of itself and acceptor proteins, which facilitates recruitment of DNA repair proteins**. In addition to its reported role in base excision repair, PARP1 plays a role in activating ATM necessary for homologous recombination and inactivating DNA-dependent protein kinase, a key component of non-homologous end-joining. PARP inhibitors directly interfere with the above functions of PARP1. In addition, PARP inhibitors have been shown to trap PARP1 on damaged DNA, leading to replication and transcription fork blockage and subsequent double-strand DNA breakage. Repair of intra/interstrand crosslinks through nucleotide excision repair or homologous recombination are also important components of the DNA repair system, and whether defects in these repair pathways can confer sensitivity to PARPi are under investigation. PARP, poly(ADP-ribose) polymerase; PARPi, PARP inhibitor; DNA polβ/δ/ε, DNA polymerase beta/delta/epsilon; XRCC1, X-ray repair cross-complementing protein 1; DNA-PKcs, DNA-dependent protein kinase catalytic subunit; KU 70/80, a.k.a XRCC6/5 (X-ray repair cross-complementing protein 6/5); ATM, ataxia telangiectasia mutated; ATR, ataxia telangiectasia and Rad3-related; γ-H2A.X, gamma-histone H2A member X; RAD51, RAD51 homolog (*S. cerevisiae*); ERCC1, DNA excision repair protein ERCC1; XPF, DNA repair endonclease XPF (xeroderma pigmentosum group F-complementing protein); FANC, Fanconi anemia.

PARPi are a class of drugs designed to compete with NAD^+^ for the substrate binding site of PARP, acting as an effective catalytic inhibitor (47). PARP inhibition has been shown to induce phosphorylation of DNA-dependent protein kinase (DNA-Pk), to further stimulate error-prone NHEJ in HR-deficient cells (44, 48, 49). More recently, another mechanism of action of PARPi involving PARP1-trapping has been proposed (50). PARPi have been shown to trap PARP1 and PARP2 while in complex with damaged DNA, resulting in cytotoxic consequences (51). Trapped PARP prevents its availability for repair function and secondarily causes replication and transcription fork blockade, and subsequent DNA breakage. This mechanism of action may be important to the clinical activity of the PARPi class. The potency in trapping PARP differs markedly among PARPi, with niraparib (MK-4827) and olaparib having greater potency than veliparib. This pattern is not correlated with the catalytic inhibitory properties of each drug. These findings suggest that PARPi have several mechanisms of action and multiple targets in the DNA repair pathway to potentially induce cancer cell death (Figure 1).

## PARP Inhibition in gBRCAm and BRCA-Like Solid Tumors

Understanding DNA repair biology has allowed the identification of patient subsets with high potential for response to PARPi treatment. The marked susceptibility of patients with gBRCAm has validated gBRCAm as a predictive biomarker for PARPi response in breast and ovarian cancer patients. In a series of pivotal preclinical studies, PARPi were noted to cause selective cytotoxicity for *in vitro* and *in vivo* models of BRCA-deficient cells (52, 53). Additionally, PARPi attenuates tumor formation in embryonic stem cell-derived teratocarcinoma xenograft models (46). These findings were translated into a phase I clinical trial of the PARPi, olaparib, in recurrent breast, ovarian, and prostate cancer patients with gBRCAm (4), initiating a new era of possibilities for the use of PARPi as single-agent therapy to treat gBRCAm-associated cancers.

The BRCA-like behavior has been described based on clinical and molecular features that parallel gBRCAm-associated cancers’ characteristics. The major clinical BRCA-like behavior identified is susceptibility to platinums and other DNA-damaging agents (54–56). Some of the molecular events described in BRCA-like behavior include epigenetic silencing of BRCA1 through promoter methylation (57–59) and overexpression of EMSY, suppressing BRCA2 transcription (60). In addition, loss or disruption of proteins necessary for HR such as RAD51, ATM, ATR, CHK1, CHK2, FANCD2, and FANCA (53, 61–64) are observed in a variety of tumors (8, 65–71), and may confer sensitivity to PARPi (8, 53). Defects in translesion synthesis (TLS) also contribute to carcinogenesis but confer sensitivity to DNA-damaging agents (72, 73), requiring further investigation on sensitivity to PARPi. Homozygous mutation in the PTEN tumor suppressor gene may also lead to HR dysfunction (74). Increased PARPi sensitivity was shown in a series of cell lines with PTEN mutation or haploinsufficiency, and confirmed in xenograft models using olaparib (74). There is also clinical evidence that olaparib may have a therapeutic utility in PTEN-deficient endometrial cancer (75, 76). Further studies are needed to investigate whether PTEN loss can serve as a potential biomarker for PARPi sensitivity (77–79). Future studies should focus on DNA profiling and the use of predictive biomarkers to select those tumors which are more likely to respond to PARPi. Ongoing research suggests HR deficiency, rather than a specific mutation in the BRCA genes, may be the main driver of cytotoxicity of PARP inhibition (45).

## Trials with PARPi in gBRCAm and/or BRCA-Like Advanced Solid Tumors

### Malignant melanoma

Little is known about the underlying cause of hereditary cancer predisposition in melanoma and its impact on the prognosis and therapeutic decisions. Cutaneous melanoma has been associated with mutations in the BRCA2 gene although there are only a few cases reported for uveal melanoma in BRCA2 mutation carriers (80). In recent years, the advent of BRAF V600E inhibitors (e.g., vemurafenib) and anti-CTLA4 antibodies (e.g., ipilimumab) has significantly improved outcomes in patients with metastatic melanoma (81–83), with a median duration of response of 8 and 16 months, respectively (84, 85). However, most patients eventually progress and some do not tolerate therapy due to immune-related side effects, indicating the need to develop other therapeutic strategies.

PARPi have multiple targets in DNA repair pathways that can potentially promote cancer cell death. In the setting of melanoma, altered expression or new mutations in DNA MMR genes, MLH1 and MSH2, have been reported in brain metastases (86). A melanoma cell line (MZ7), derived from a patient who received dacarbazine therapy, exhibited a high level of resistance to temozolomide (TMZ) without expressing *O*(6)-methylguanine-DNA methyltransferase (MGMT), which was related to impaired expression of MSH2 and MSH6 (87). PARP inhibition with INO-1001 has been shown to restore sensitivity to TMZ in an MMR-deficient xenograft model of malignant melanoma (88), and another PARPi, GPI 15427, enhanced TMZ anti-tumor activity in various cancers, including metastatic melanoma in an orthotopic xenograft mouse model (24). These preclinical studies provide evidence that MMR loss of function is a potential predictive biomarker of PARPi responsiveness in patients with metastatic melanoma.

A number of clinical trials of PARPi in melanoma patients have been conducted or are ongoing although they have not specifically addressed the frequency of HR dysfunction/gBRCAm in their populations. Bedikian et al. reported the results of a phase IB study of intravenous INO-1001 and oral TMZ in unselected patients with unresectable stage III or IV melanoma (89). The dose limiting toxicities (DLTs) were elevation of liver transaminases and myelosuppression at the 400-mg dose of INO-1001. Of the 12 patients enrolled, 1 patient had a partial response (PR) and 4 patients had stable disease (SD). Several phase II studies using PARPi either as a single-agent or in combination with chemotherapy, radiotherapy, or targeted therapy are summarized in Table 3. A phase II trial sought to evaluate the combination of rucaparib and TMZ in patients with metastatic malignant melanoma (90). The disease-control rate was 40% (8/20), where four patients attained a PR and four others had prolonged SD. In total, 12 of the 40 patients required a dose reduction of TMZ secondary to myelosuppression (90). Another phase II study evaluated treatment with rucaparib 12 mg/m^2^ and TMZ 200 mg/m^2^ in patients with advanced melanoma. Myelosuppression was again noted, with 25 patients (54%) requiring a 25% dose reduction in TMZ. The RR was 17.4%, with median time to progression and OS of 3.5 and 9.9 months, respectively. This study demonstrated that TMZ could safely be given with a PARP-inhibitory dose (PID) of rucaparib (12 mg/m^2^), based on 74–97% inhibition in PARP of peripheral blood mononuclear cells (PBMCs). This resulted in an increase in PFS compared with historical controls (91). Phase I and II trials evaluating E7016 in combination with TMZ in patients with advanced solid tumors and malignant melanoma are ongoing (92, 93). Eligibility criteria for the phase II study include BRAF wild-type status and no prior treatment with TMZ or PARPi. As substantial progress has been made in the management of malignant melanoma in recent years (94), it remains to be seen whether PARPi will be added to the treatment armamentarium.

### Pancreatic cancer

Hereditary pancreatic cancer is rare and extremely heterogeneous, and it accounts for approximately 2% of all pancreatic cancer cases. The major component of hereditary pancreatic cancer is the familial pancreatic cancer syndrome. Although up to 20% of hereditary pancreatic cancer cases are associated with germline mutations in BRCA2, CDKN2A, PRSS1, STKI1, or MMR genes, the major underlying gene defects are still unknown (95). BRCA2 mutation prevalence in familial pancreatic cancer patients varies between 5 and 19% (30), and a BRCA2 mutation increases the risk of developing pancreatic cancer by approximately 3.5-fold (96). The unique biology of cancer cells with BRCA mutations offers potential therapeutic advantages with agents such as platinums. However, one case series report patients with gBRCAm did not reveal a benefit to first line platinum chemotherapy in the treatment of advanced pancreatic cancer (97), although this needs to be further evaluated in a selected study for pancreatic cancer with gBRCAm. Preclinical studies have shown single-agent activity of PARPi (98), as well as radiosensitization in combination with chemoradiation in BRCA2-deficient pancreatic cells (25). Studies are ongoing to examine single-agent and combination PARPi therapy in BRCA2 mutant pancreatic cancers.

Interim results from an ongoing phase II study of olaparib monotherapy in gBRCAm-associated advanced solid cancers were recently reported (99). Nearly 8% of the patients (23/298) had advanced/recurrent pancreatic cancer. A RR of 5/23 (21.7%) was noted, with eight patients achieving SD. This yielded a clinical benefit rate of 57% in gBRCAm-associated pancreatic cancer patients. Pishvaian et al. reported a phase I study of veliparib with concurrent FOLFOX chemotherapy in patients with metastatic pancreatic cancer (100). Twenty-eight patients were enrolled in the trial and at the time of review, data were available for 18 patients. For the 11 patients who were treated in the first line setting, RR was 18%, with a PFS and OS of 3.9 and 7.4 months, respectively (Table 3). Therefore, the investigators concluded that the experimental combination regimen could be given safely, and was modestly active (100). These data support further evaluation of PARPi either as different combinations or more potent PARPi with chemotherapy and/or other targeted agents combination in this subgroup of pancreatic cancer patients.

### Prostate cancer

Germline BRCA2 mutation confers the highest genetic risk of prostate cancer known to date at 8.6-fold in men ≤65 years, whereas the effect of BRCA1 is more modest at 3.4-fold (32, 33, 101, 102). Prostate cancer in patients with gBRCAm tends to be more aggressive, with a higher likelihood of nodal involvement and distant metastasis with inferior survival outcomes (103). Trials analyzing the response of these patients to DNA-damaging agents, such as platinums, and identifying the therapeutic targets of this subgroup are urgently needed.

Single-agent olaparib has demonstrated activity in patients with gBRCAm castration resistant prostate cancer (CRPC). A phase I olaparib study by Fong et al. reports one gBRCA2m patient treated with single-agent olaparib who sustained a CR lasting in excess of 2 years (4). Recently, Sandhu et al. presented clinical data on four patients with advanced gBRCAm CRPC, three of whom were treated with olaparib and one with niraparib (104). Two patients on olaparib showed prostate-specific antigen (PSA) and radiologic responses lasting 26 and 34 months, respectively, while the third patient had SD for 10 months. The patient on niraparib exhibited primary resistance with development of a new liver lesion and a rise in PSA of nearly threefold at the time of the first reassessment. Translational studies revealed positive ERG staining by immunohistochemistry, and ERG rearrangements by FISH, as well as either heterozygous or homozygous PTEN allelic loss in all four cases. Subsets of patients with CRPC are also known to manifest increased PARP activity (105). This potentially opens another avenue for therapy utilizing PARPi, although gBRCAm is a very rare event in prostate cancer.

Gene fusion between the ERG proto-oncogene and TMPRSS2 promoter is a major genomic alteration observed in approximately 50% of prostate cancers. Formation of the TMPRSS2-ERG fusion gene causes aberrant androgen-dependent ERG expression (106) and promotes tumorigenesis (107). Preclinical studies have shown that PARP1 directly interacts with ERG to inhibit ETS gene fusion protein activity. In turn, inhibition of PARP1 reduces ETS-positive, but not ETS-negative, prostate cancer xenograft growth (108). This may be a useful predictive biomarker for PARPi sensitivity.

Other preclinical studies include radiosensitization by rucaparib, most evident in PTEN-deficient prostate cancer cells containing the TMPRSS2-ERG fusion gene (109). However, no association was noted between loss of PTEN expression by immunohistochemistry and ETS rearrangements by FISH, with radiologic assessment of the anti-tumor activity of niraparib in 18 patients with prostate cancer (110). The HR/PARP synthetic lethality model may be more widely applicable in prostate cancer with germline or somatic inactivating mutations in the HR DNA repair genes, CHK2, BRIPI/FANCJ, NBS1, BRCA1, and ATM, collectively thought to occur in 20–25% of prostate cancer cases. Recently, a phase II study of olaparib in unselected patients with CRPC was initiated (111).

Veliparib has also been investigated and shown to enhance the anti-tumor activity of TMZ in prostate cancer xenografts, yielding tumor size reduction in TMZ-resistant PC3-Leu prostate cancer mice (112). This formed the rationale for testing the efficacy and safety of veliparib and TMZ in 26 patients with metastatic CRPC (113). Grade III/IV thrombocytopenia was noted in 15% of patients. Two patients had a confirmed PSA response and four patients had SD for at least 4 months. The median PFS and OS were 2.1 (95% CI: 1.8, 3.9) and 9.1 (95% CI: 5.5, 11.7) months, respectively. This study suggested veliparib and TMZ are tolerated well, but with limited clinical activity. Future trials will explore the use of different chemotherapy agents in combination with higher doses of veliparib. Overall, further evaluation of biochemical changes or predictive biomarkers in response to PARPi in advanced prostate cancer is needed.

### Colon cancer

Preclinical data suggest the utility of PARPi in tumors deficient in HR and displaying microsatellite instability (MSI) due to mutations in the coding microsatellites of the MRE11A and hRAD50 genes involved in DNA DSB repair (114). Preferential cytotoxicity to the PARP1 inhibitor ABT-888 was seen in MSI cell lines containing mutant copies of MRE11A, compared with wild-type or microsatellite stable (MSS) cells (115). In a recent study, the observed ability of MSH3 to protect against DSB was exploited by the combination of oxaliplatin and a PARPi, which produced a synergistic cytotoxic effect against CRC cells (116). Another study reporting high correlation between MRE11 mutations and MSI in CRC cell lines as well as primary tumors, found that PARPi preferentially kills MSI cell lines harboring MRE11 mutations (115). The data suggest a role for PARPi in MSI-CRC treatment, providing a rationale for clinical studies in this subset of patients.

Dozens of potential PARPi have been screened *in vitro* and *in vivo* to select candidates for clinical evaluation as a chemosensitizer in CRC (117). A phase II trial is currently evaluating the efficacy of olaparib in metastatic CRC (mCRC) stratified for MSI status (118). Twenty-two patients with MSI-negative tumors were enrolled and received a mean number of two cycles. Preliminary data indicate no single-agent activity of olaparib against non-MSI-high (MSI-H) mCRC. Accrual of MSI-H mCRC patients continues, along with active biomarker analysis. Other clinical trials of PARPi in MSI-CRC are in progress.

Studies have evaluated and validated veliparib as a sensitizer to irinotecan, oxaliplatin, and radiation therapy (RT) in CRC cells (26, 119). Several phase II studies are evaluating the role of PARPi as a chemosensitizer in patients with advanced and mCRC, irrespective of MSI status (Table 2). Pishvaian et al. (120) conducted a single arm, open label phase II study in patients with unresectable or mCRC. Patients were treated with TMZ (150 mg/m^2^ orally daily) days 1–5, and veliparib (40 mg orally twice a day) days 1–7 of each 28-day cycle. Immunohistochemistry was performed on archived tumor samples to quantify MMR and PTEN protein expression. The combination of veliparib and TMZ was well tolerated in the 47 patients treated, with a disease-control rate of 23%. The results of immunohistochemistry for the MMR and PTEN proteins from 45 archived tumor samples are not yet reported. It was concluded that, in a heavily pre-treated population of patients with mCRC, the combination of veliparib and TMZ can be safely given, and displayed limited clinical activity.

### Lung cancer

Reduced BRCA1 mRNA and protein expression levels have been observed in up to 44% of NSCLC, occurring through various mechanisms such as promoter hypermethylation (121). One study showed that BRCA1 silencing increased susceptibility to olaparib treatment in NSCLC cell lines (122), providing evidence for possible clinical application in this subset of NSCLCs. A future study will assess the utility of olaparib in delaying the time to disease progression in patients with advanced NSCLC who have responded to initial chemotherapy (123). The role of PTEN mutation and its effect on the susceptibility to PARPi is an area of continued research in lung and other malignancies. Up to 9% of NSCLCs have a somatic mutation in PTEN. Olaparib has yielded additive activity with cisplatin in homozygous deleted PTEN-deficient NSCLC cells and xenograft models (79). Another gene involved in DNA repair, excision repair cross-complementation group 1 (ERCC1), is a key component of NER and the main mechanism for removing platinum–DNA adducts (124). Preclinical studies have explored this repair pathway, demonstrating synergy of olaparib and veliparib with cisplatin in NSCLC cell lines with low ERCC1 expression levels (125, 126). PARPi have also been explored preclinically in combination with other DNA-damaging modalities such as RT (16).

The role of PARPi in patients with EGFR mutant NSCLC has been studied in a phase IB study of olaparib and the EGFR tyrosine kinase inhibitor (TKI) gefitinib (127). It was noted that high BRCA1 mRNA expression is associated with a shorter PFS in EGFR-mutated patients treated with erlotinib. To date, 18 patients have received treatment at four different dose levels of olaparib ranging 100–200 mg twice daily dose, and 200–250 mg three times daily dose. DLT was grade 3 anemia observed at dose level 4 (250 mg three times daily). Of the 17 patients in whom a disease response could be evaluated, 7 (41.1%) had a PR. All of the patients who responded were EGFR TKI naive. Another seven patients (41.1%), most of whom received prior treatment, had documented SD, and three patients (17.6%), all of whom had prior EGFR TKI treatment, progressed. The observed anti-tumor activity will be further evaluated in EGFR TKI treatment-naive patients with EGFR-mutated NSCLC; a phase II randomized trial comparing the efficacy of olaparib and gefitinib versus gefitinib alone was launched in July 2013.

Multiple studies are also exploring the role of PARPi in combination with chemotherapy and/or RT in NSCLC. A phase I dose-escalation trial of olaparib and concurrent RT, with or without cisplatin, is ongoing in patients with advanced NSCLC (128). SWOG 1206, a phase I/II trial, is evaluating the use of veliparib with or without RT and carboplatin/paclitaxel in patients with inoperable stage III NSCLC. Several similar studies involving other combinations of PARPi ± chemotherapy and/or RT are ongoing in patients with NSCLC (Table 2). Ultimately, combining PARPi with cisplatin or radiotherapy may prove to be a useful strategy in the treatment of NSCLC.

### Ewing’s sarcoma

PARPi has preclinically shown anti-tumor activity in the treatment of Ewing’s sarcoma. Gene fusions involving Ewing’s sarcoma breakpoint region 1 (EWS) and ETS transcription factors have been implicated in abnormal proliferation, invasion, and tumorigenesis (129). PARP inhibition has been evaluated as an effective treatment option for Ewing’s sarcoma with EWS–FLI1 or EWS–ERG genomic fusions in xenograft models (130), and olaparib has been shown to have potent activity in cell lines with a EWS/FLI1 translocation (131). Additionally, a study in preclinical models showed synergy between PARPi and TMZ (130) in the treatment of Ewing’s sarcoma cell lines. Currently, a number of clinical trials investigating the utility of PARPi in Ewing’s sarcoma are underway (132, 133).

## Challenges and Future Directions for Clinical Development in Cancers Other than Breast and Ovarian

There is considerable interest in the clinical development of PARPi for use in solid tumors other than breast and ovarian cancers. The optimal dose, scheduling, and sequencing of PARPi, and combination with other cytotoxic or biologic agents need to be evaluated in carefully designed clinical trials. The incorporation of predictive biomarkers into studies of gBRCAm and BRCA-like cancers presents challenges. First is the development of a mechanism with which to identify patients who are most likely to benefit from PARPi therapy. Predictive biomarkers applied to readily available bioresources, such as archival tissue or non-tumor tissue, have been proposed. Changes in or baseline PAR incorporation into PBMC DNA was suggested and evaluated as a putative early on-treatment pharmacodynamic measure; while present, there was no relationship to clinical outcome (134). BRCA1/2 somatic mutation or promoter methylation, ATM mutation, MRE11-dominant negative mutations in MMR-deficient cancers, FANCF promotor methylation and PTEN deficiency are all potential biomarkers of sensitivity to PARPi (51). Importantly, not all patients with deficiencies in BRCA1 or 2 are responsive to PARP inhibition (135). Therefore, identification and validation of predictive biomarkers of those gBRCAm who will respond to PARPi is also an important area of ongoing research.

The second challenge is dissecting and defining mechanisms of development of resistance to PARPi, and whether they portend potential collateral resistance to other DNA-damaging agents. Acquisition of a secondary mutation in BRCA1/2 that allows BRCA1/2 gene read-through and yields a functional protein has been demonstrated in cell lines and some patients; this was correlated with loss of susceptibility to PARPi treatment (136). Other potential mechanisms of clinical resistance have been proposed based on preclinical models, including loss of 53BP1, or increased activity of RAD51 (137, 138). Whether these findings can be used as selective or predictive biomarker is yet to be determined. Ang et al. recently reported that gBRCAm-associated ovarian cancer patients retain the potential to respond to subsequent chemotherapy, including platinum-based agents, after progression on PARPi (139). This observation has implications for chemotherapy sequencing. Further studies are needed to evaluate outcomes to subsequent chemotherapies or another PARPi in other solid tumor patients who have a BRCA-like phenotype. Understanding the mechanism(s) of resistance to PARPi will lead to optimal application and sequencing of PARPi and other DNA-damaging agents.

## Conclusion

PARPi are a class of agents with mechanisms of action beyond their documented role in BER pathway. They potentially have a broader application in the treatment of cancer patients, both within the confines of gBRCAm and BRCA-like disease, but also extending to a wide range of aberrations in DNA damage repair pathways. Ongoing research will aim to identify optimal predictive biomarkers in order to improve patient selection and thus, clinical responses to treatment. It is anticipated that novel clinical trial design strategies will help minimize toxicity and maximize therapeutic efficacy. Other pertinent questions relate to the duration of treatment and long-term effects of treatment, which need to be carefully investigated (20). Future directions for PARPi will include clinical trials directed at patient subsets that are most likely to respond to treatment, based on their molecular characteristics and predictive biomarkers. This may ultimately result in practice-changing treatments in malignancies such as pancreatic cancer, prostate cancer, and Ewing’s sarcoma. The results of trials of PARPi, either as single-agents or in combination with chemotherapy, RT, or biological agents in other solid tumors are eagerly awaited.

## Author Contributions

All authors substantially contributed to the concept of the manuscript, drafted and revised, and approved final version.

## Conflict of Interest Statement

The authors declare that the research was conducted in the absence of any commercial or financial relationships that could be construed as a potential conflict of interest.
